# A retrospective study of acute pancreatitis in patients with hemorrhagic fever with renal syndrome

**DOI:** 10.1186/1471-230X-13-171

**Published:** 2013-12-17

**Authors:** Yin Zhu, You-Xiang Chen, Yong Zhu, Pi Liu, Hao Zeng, Nong-Hua Lu

**Affiliations:** 1 Department of Gastroenterology, The First Affiliated Hospital of NanChang University, Nanchang, Jiangxi 330006, People’s Republic of China

**Keywords:** Etiology, Acute pancreatitis, Hemorrhagic fever with renal syndrome

## Abstract

**Background:**

Etiological diagnosis is an important part of the diagnosis and treatment of acute pancreatitis. Hantavirus infection is a rare cause of acute pancreatitis, which is easy to ignore. There is a need to analyze clinical features of acute pancreatitis caused by Hantavirus.

**Methods:**

This is a retrospective study conducted from May 1, 2006 to May 31, 2012 on patients diagnosed with hemorrhagic fever with renal syndrome at our hospital. We reviewed these patients medical records, laboratory results and radiologic examinations to determine the prevalence and summarize clinical features of acute pancreatitis in patients with hemorrhagic fever with renal syndrome.

**Results:**

A total of 218 patients were diagnosed with hemorrhagic fever with renal syndrome during the 6-year study period. Only 2.8% (6/218) of the total hemorrhagic fever with renal syndrome patients were diagnosed with acute pancreatitis. The first symptom for all six of the patients with acute pancreatitis was fever. All six patients experienced hemorrhage and thrombocytopenia during the disease course, which was different from general acute pancreatitis. In addition, we presented two misdiagnosed clinical cases.

**Conclusions:**

Acute pancreatitis is not a frequent complication in patients with hemorrhagic fever with renal syndrome. Clinicians should be alerted to the possibility of hemorrhagic fever with renal syndrome when acute pancreatitis patients with epidemiological data have high fever before abdominal pain.

## Background

The incidence of acute pancreatitis (AP) continues to rise worldwide, with the current annual numbers of new cases ranging from 5-80/100,000 for different countries. The most frequently implicated etiologies are gallstones and alcohol abuse [1]. Viral pathogens, such as Coxsackie virus, human immunodeficiency virus (HIV) and Hantavirus, are relatively rare causes of acute pancreatitis in humans, which is easy to ignore. Hantavirus infection is more common in Asia and Europe and can manifest as hemorrhagic fever with renal syndrome (HFRS) [2]. The clinical features of HFRS are diverse, with hemorrhage, fever, thrombocytopenia, and acute renal insufficiency frequently observed and considered clinical hallmarks of the disease [3]. Although it has been reported that a large portion of HRFS patients (64.4%) present with a complaint of abdominal pain, AP is still a rare complication of HFRS [2]. However, some clinicians think that AP in patients with HFRS is much more common than previously recognized [4].

Here, we describe our findings from a retrospective review of our hospital’s patients with HFRS that was carried out to determine the incidence of associated acute pancreatitis. In addition, we present a series of previously misdiagnosed clinical cases, followed by a comprehensive review of the publicly available literature.

## Methods

### Patient selection

Hospital records, including medical records, laboratory results and radiological examinations, from May 1, 2006 to May 31, 2012 were reviewed to identify all patients having a discharge diagnosis of HFRS. Patients were selected for study according to documented symptoms and signs compatible with HFRS, and Hantavius infection confirmed by serological evidence (positive enzyme-linked immunosorbent assay (ELISA) tests for immunoglobulin IgM or IgG antibodies to Hantavirus).

The studies received the approval of the ethics committee of the First Affiliated Hospital, Nanchang University.

### AP diagnosis

AP diagnosis was made according to the presence of at least two of the following three features [5]: (a) abdominal pain suggestive of pancreatitis (epigastric pain often radiating to the back), with the start of such pain considered to be the onset of acute pancreatitis; (b) serum amylase and lipase levels three or more times above the normal range; and (c) characteristic findings on computed tomography (CT) and/or magnetic resonance images (MRI), or by transabdominal ultrasonography (US). Mild acute pancreatitis (MAP) and severe acute pancreatitis (SAP) was defined according to the Atlanta criteria [5].

### Statistical analysis

The χ2 test was performed, with a 95% confidence interval, to compare the fatality rates between HFRS patients with pancreatitis and without pancreatitis. Intergroup differences were considered significant when the *p*-value was less than 0.05. All statistical analyses were carried out with SPSS software (v17.0; SPSS Inc., Chicago, IL, USA).

## Results

A total of 218 patients were diagnosed with HFRS at our hospital during the 6-year study period. These patients comprised 150 males and 68 females (male-to-female ratio: ~2:1) between 11 and 75 years old (average age: 43.5 ± 10.0 years). Only 2.8% (6/218) of the total HFRS patients were diagnosed with AP, including two males and four females (1:2) between 22 and 48 years old (33.0 ± 7.7). All six AP patients had positive ELISA results for both IgM and IgG antibodies to Hantavirus. Two of the six had SAP, and the remaining four had MAP.

Five (83.3%) of the HFRS-AP patients complained of constant upper abdominal pain, while another patient experienced lumbago. In general, upper abdominal pain was present from the onset of the first day to the fifth day (2.8 ± 1.6 days). All six patients had increased serum amylase, and four (66.7%) showed results that were three or more times above the normal range at least once during their hospitalization. The first symptom for all six of the patients was reported to be fever. All six patients experienced clinically observed hemorrhage and thrombocytopenia during the disease course. Five of the patients were diagnosed with acute renal insufficiency.

Upon discharge, all six of the HFRS-AP patients were reported as cured and none died during the disease course. Of the 212 HFRS patients without AP, 172 (81.1%) were reported as cured upon discharge. Fourteen (6.6%) patients died during the disease course in-hospital. The difference in fatality rates between the HFRS groups with AP and without AP did not reach statistical significance (*p* > 0.05).

Both of the HRFS-SAP patients were misdiagnosed during the in-hospital disease course. These cases are detailed below in order to improve the understanding of HFRS associated AP.

### Case report 1

A 41-year-old male presented to the Department of Gastroenterology complaining of 24 hours of upper abdominal pain, high fever, and diarrhea. On admission, physical examination revealed a mild fever (37.6°C), mildly hypertensive blood pressure (145/99 mmHg), and pulse and respiration rates on the upper end of normal (96/min and 20/min, respectively). The patient’s abdomen was obviously distended and abdominal muscles were tense with apparent tenderness and rebound tenderness.

The bowel sounds were also decreased. The patient had a history of cholecystolithiasis, but no history of alcohol abuse or medication.

**Figure 1:**
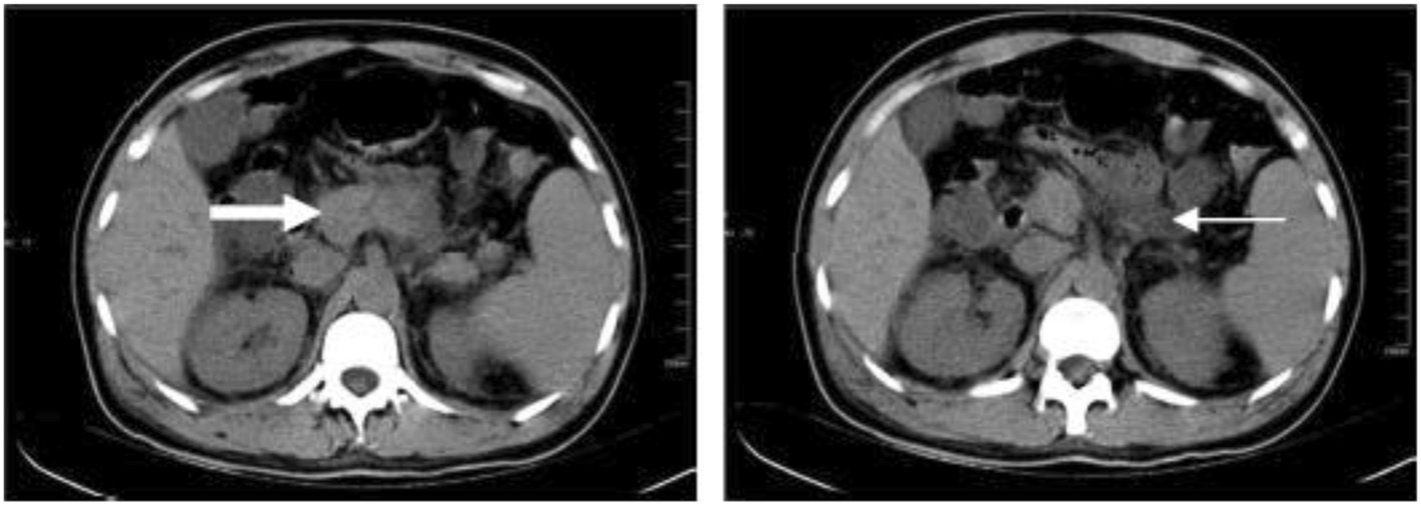
**Abdominal CT images of a 41 year-old man with HFRS complicated with SAP (case 1).** Edema of the pancreas (thick white arrow) and peripancreatic fluid collection (thin white arrow) are visible.

Routine laboratory tests were ordered. Blood tests for inflammation were positive, showing elevated C-reactive protein (CRP; 63.9 mg/L vs. normal range: 0-8 mg/L), leukocytosis (25.4 × 10^9^/L vs. 4-10 × 10^9^/L), and elevated neutrophils (21.3 × 10^9^/L vs. 2.0-8.0 × 10^9^/L). The platelet count was low (32 × 10^9^/L vs. 100-300 × 10^9^/L). Pancreatic enzymes were normal, with serum amylase being 48 U/L (0-103 U/L) and serum lipase being 17 U/L (0-60 U/L). However, tests of renal function markers indicated impairment, with increased serum creatinine (226 μmol/L vs. 44-106 μmol/L) and increased blood urea nitrogen (BUN; 19.7 mmol/L vs. 2.3-7.8 mmol/L). In addition, liver enzyme analyses indicated impaired hepatic function, with elevated alanine aminotransferase (ALT; 80 U/L vs. 0-40 U/L) and aspartate aminotransferase (AST; 156 U/L vs. 0-40 U/L). However, both total bilirubin and direct bilirubin were normal. Albumin was decreased (28.7 g/L vs. 35-55 g/L). The prothrombin time was normal (13.7 s vs. 10-13 s), but the activated coagulation time of whole blood was elevated (102.5 s vs. normal range: 23.7-36.4 s). Both the fecal and urine occult blood tests were positive. All other routine laboratory values were within the normal range. CT images of the thoraco–abdominal region revealed small pleural effusions, seroperitoneum, pancreatic edema, peripancreatic fat stranding, and stones in the gallbladder (Figure 1). MR cholangiopancreatography also showed gallbladder stones, but the bile duct was not dilated. Based on these findings, the patient was initially diagnosed with gallstone-induced SAP. The patient was immediately given fluid resuscitation, fresh frozen plasma, antibiotics, and somatostatin. Hemofiltration was initiated upon detection of azotemia. Three days after hospital admission, the patient exhibited facial flushing, conjunctival injection, and polyuria. The treating physician sought to reassess the patient’s medical history and upon additional questioning, the patient revealed that he had experienced a high fever with headache and orbital ache 4 days prior to presenting to the hospital. The physician ordered an ELISA test for IgM and IgG antibodies of Hantavirus, and the test results were positive. On the fifth day of hospitalization, the patient’s serum amylase level was retested and found to have become significantly elevated (by nearly 10-fold, to 474 U/L). The patient was diagnosed with HFRS complicated by SAP.

On the seventh day of hospitalization, the patient’s condition necessitated enteral nutrition *via* a fine-bore naso-jejunal tube. Over the following days, the patient’s laboratory values gradually returned to within normal ranges. On the fifteenth day, the patient was discharged with full recovery. At the 5-month follow-up, the patient showed no signs or symptoms of HFRS or SAP.

### Case report 2

A 22-year-old female was admitted to the Intensive Care Unit (ICU) of our hospital with a history of high fever lasting 1 week and severe upper abdominal pain with nausea, vomiting, and diarrhea. The vomitus contained a small amount of red fluid and her stools were often dark. On admission to the ICU, physical examination revealed a normal temperature (36.6°C), normal blood pressure (115/70 mmHg), high pulse rate (115/min), and respiration rates on the upper end of normal (20/min). The patient showed signs of confusion, conjunctival injection, and sclera xanthochromia.

All of the patient’s abdominal muscles were tense with apparent tenderness, and the bowel sounds were decreased. There was also a significant amount of dark red fluid coming from the vagina, but the patient was not currently menstruating. The patient had no history of alcohol abuse, medication, or biliary tract disease.

Laboratory tests performed upon ICU admission provided the following results.

Hemocyte analysis indicated a systemic inflammatory state, with leukocytosis (20.9 × 10^9^/L vs. 4-10 × 10^9^/L) and elevated neutrophils (14.2 × 10^9^/L vs. 2.0-8.0 × 10^9^/L). The platelet count was low (12 × 10^9^/L vs. 100-300 × 10^9^/L) and the hemoglobin was low (76 g/L vs. 110-170 g/L), but serum amylase was slightly elevated (120 U/L vs. 0-103 U/L). Tests of renal function indicated impairment, as shown by elevated serum creatinine (362 μmol/L vs. 44-106 μmol/L) and BUN (29.9 mmol/L vs. 2.3-7.8 mmol/L). In addition, liver function tests indicated impairment, with substantially elevated levels of ALT (262 U/L vs. 0-40 U/L) and AST (1359 U/L vs. 0-40 U/L). Total bilirubin was also high (37.8 μmol/L vs. 0-20 μmol/L), as was direct bilirubin (21.8 μmol/L vs. 0-7 μmol/L), but albumin was low (31.5 g/L vs. 35-55 g/L). Creatine kinase was elevated (353 U/L vs. 20-170 U/L), as was the creatine kinase myocardium isozyme (75 U/L vs. 0-20 U/L). In addition, the patient was hyperglycemic (blood glucose: 7.5 mmol/L vs. 3.8-6.1 mmol/L). Both the prothrombin time (22.2 s vs. 10-13 s) and activated coagulation time of whole blood (74.4 s vs. 23.7-36.4 s) were increased. Urine analysis showed proteinuria, hematuria, and occult blood positivity. While the serum sodium level was normal (125 mmol/L vs. 135-145 mmol/L), the serum calcium level was slightly low (1.72 mmol/L vs. 2.0-2.6 mmol/L). All other routine laboratory values were within the normal range.

**Figure 2:**
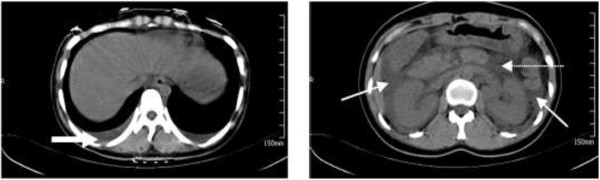
**Thoraco–abdominal CT images of a 22 year-old woman with HFRS complicated with SAP (case 2).** Pleural effusions (thick white arrow), peripancreatic fluid collection (thin white arrow), and peripancreatic fat stranding (dotted-line white arrow) are shown.

CT images of the thoraco–abdominal region revealed bilateral pleural effusions, seroperitoneum, pancreatic edema, peripancreatic fat stranding, and a slightly enlarged spleen (Figure 2).

Based on the above findings, the initial diagnosis was multiorgan dysfunction syndrome with SAP and uterine hemorrhage. The patient was treated in the ICU with blood transfusion including erythrocyte suspension, and was given platelets, fresh frozen plasma, etamsylate, antibiotics, somatostatin, omeprazole, and reduced glutathione. On the third day of ICU hospitalization, the patient’s condition had improved and her symptoms of high fever, abdominal pain, and uterine hemorrhage had become significantly relieved or completely resolved. The patient was transferred to the Department of Gastroenterology for further treatment to address the SAP. On the third day of hospitalization in the Department of Gastroenterology, the patient developed polyuria and her serum amylase level had become substantially elevated (416 U/L vs. 0-103 U/L). The treating physician considered alternative causes of SAP since the patient lacked signs of the common causative factors, including gallstones, alcohol abuse, and hyperlipidemia. The physician ordered an ELISA test of the IgM and IgG antibodies of Hantavirus and the result was positive. The patient’s diagnosis was corrected to HFRS complicated with SAP.

On the seventh day of hospitalization, a fine-bore naso-jejunal tube was inserted to provide enteral nutrition. All of the patient’s laboratory values gradually returned to within the normal ranges over the following 3 days. On the tenth day, the patient was discharged with full recovery. At the 1-year follow-up, the patient showed no signs or symptoms of HFRS or SAP.

## Discussion

HFRS shows distinctive clinical manifestations throughout the disease course, from acute influenza-like febrile illness to shock syndrome. The key feature of all stages is renal involvement; however, several extrarenal manifestations have also been reported, including acute visual impairment, acute myopia, central nervous system complications with seizures, myocarditis, severe gastrointestinal hemorrhaging, and abdominal pain. Unfamiliarity with these less frequent extrarenal manifestations may complicate diagnosis upon clinical presentation, leading to misdiagnoses, administration of unnecessary treatments and procedures, and delay in time to treatment [2].

Abdominal pain is a common symptom of Hantavirus infection, with an estimated 46% of patients presenting with this as their initial complaint or experiencing it at some time during their hospitalization [6]. The pathogenesis of Hantavirus infection is well-studied, and one of the key features is increased vascular permeability [4]. During the infection, the patient’s capillaries become engorged and focal hemorrhages develop; ultimately, the systemic expansion of capillary leakage leads to retroperitoneal edema, which may affect the pancreas[2]. Interestingly, the pancreas appeared unremarkable upon gross examination but microscopic examination revealed mild interstitial hemorrhage and vascular congestion[5]. These are the possible pathogenic mechanisms of acute pancreatitis in HFRS. Therefore, Hantavirus is believed to induce life-threatening effects on a microscopic level, whereby the body’s normal physiological processes are at first disrupted but eventually develop into full organ failure.

A search of the PubMed literature database found 5 clinical reports of HFRS complicated with AP, comprising 17 patients. Bui-Mansfield *et al*. [6] reported 13 cases of HFRS, seven of which (53.8%) were complicated with AP; the authors suggested that this high proportion may indicate that AP in patients with HFRS may be much more common than previously recognized. In a recent study, Park *et al*. [2] analyzed the incidence of extrarenal manifestations of HFRS in 73 patients and found that the most common symptom was fever (94.5%), followed by abdominal pain (64.4%) and headache (50.7%). Furthermore, they found that the most common physical signs were conjunctival hemorrhage (74.0%), flank tenderness (56.2%), and petechial (56.2%). Of the 73 HFRS patients in their study, only eight manifested pancreatobiliary involvement, and the manifestations were rather heterogeneous: acalculous cholecystitis (n = 4, 5.6%), pancreatitis (n = 3, 4.1%), and cholangitis (n = 1, 1.4%). In general, the patients with pancreatobiliary manifestations showed elevated pancreatic enzymes and liver function tests. Thus, it was recommended that HFRS patients with abdominal pain and abnormal pancreatic or liver enzyme levels be subjected to further imaging analyses, including CT scans or US, to determine the presence and severity of pancreatobiliary complications[2]. Our retrospective study of 218 HFRS patients indicated that only six (2.75%) were complicated with AP. It suggests that prevalence of HFRS complicated with AP was low, which was similar to that reported by Park *et al.*[2]. Our literature search identified two case reports of patients with HFRS complicated with AP. The authors suggested that one cause of abdominal pain in patients with HFRS may be acute pancreatitis [3,4]. In fact, many patients with HFRS do complain of abdominal pain. We suggest that pancreatic enzymes should be monitored in HFRS patients with abdominal pain, especially in those patients with constant upper abdominal pain radiating to the back. As shown by the two cases presented herein, pancreatic enzymes may be normal or slightly increased at the onset of abdominal pain, but may significantly increase after several days. Liem *et al.*[6] reported that patients with HFRS and associated pancreatitis had increased morbidity, but the difference from the control group did not reach statistical significance. In our series of six HFRS-AP patients, all responded to the in-hosptial therapeutic interventions and were discharged with full recovery. We did note, however, that the fatality rate was higher in the HFRS patients without AP than in the patients with AP, but the difference was not statistically significant (6.6% vs 0% *p* > 0.05). This finding suggests that the complicating AP did not increase the fatality rate of HFRS.

Fever is seldom a feature of AP in the first day after onset. If present, fever is usually considered an indicator of potential cholangitis [1]. All of the six HFRS-AP patients from our hospital experienced high fever prior to the occurrence of abdominal pain.

One of these six patients had a history of cholecystolithiasis, and the treating physician had mistakenly presumed that the symptom of high fever was due to biliary tract infection. This was one of the features that led to the patient receiving an initial misdiagnosis of gallstone-induced SAP. It was not until the patient developed facial flushing, conjunctival injection and polyuria that the conditions of high fever, thrombocytopenia and acute renal insufficiency were considered with more emphasis and the patient was properly diagnosed with HFRS complicated with AP. All six patients experienced hemorrhage and thrombocytopenia during the disease course, which is a distinguishing feature from general acute pancreatitis. This case highlighted the importance of confirming the underlying cause of a high fever experienced before abdominal pain in AP patients. The HFRS-AP cases in our hospital series, especially the two with initial misdiagnosis, highlight the importance of identifying and confirming the etiology of AP. Although AP is seldom caused by the infection of Hantavirus, it should be considered if AP patients with acute renal insufficiency have high fever before abdominal pain especially during the prevailing season in the epidemic areas of HFRS. In addition, when HFRS patients present with abdominal pain, pancreatic enzyme levels should be detected and imaging analyses should be conducted to assess the function and morphology of the pancreas to detect the complications of AP.

## Conclusions

Our study has shown that HFRS is seldom complicated with AP and it does not increase the fatality rate of HFRS. It is important to confirm the etiology of AP. Clinicians should be alert to the possibility of HFRS when AP patients with epidemiological data have acute renal insufficiency and high fever before abdominal pain.

### Consent

Written informed consent was obtained from the two patients whose cases are discussed in detailed for publication of clinical details and any accompanying images. A copy of the written consent is available for review by the Editor of this journal.

## Abbreviations

AP: Acute pancreatitis; HIV: Human immunodeficiency virus; HFRS: Hemorrhagic fever with renal syndrome; ELISA: Enzyme-linked immunosorbent assay; CT: Computed tomography; MRI: Magnetic resonance images; US: Ultrasonography; MAP: Mild acute pancreatitis; SAP: Severe acute pancreatitis; CRP: C-reactive protein; BUN: Blood urea nitrogen; ALT: Alanine aminotransferase; AST: Aspartate aminotransferase; ICU: Intensive care unit.

## Competing interests

The authors have no any potential competing interests.

## Authors’ contributions

ZY obtained and analyzed the data and wrote the manuscript; CYX conceived of the study, and participated in its design; ZY, LP, and ZH obtained and interpreted data and participated in its design; LNH made substantial contributions to conception, design and coordination of the study and gave final approval of the version to be published. All authors read and approved the final manuscript.

## Pre-publication history

The pre-publication history for this paper can be accessed here:

http://www.biomedcentral.com/1471-230X/13/171/prepub
